# *Augenblickdiagnose* in neurology: Revisiting ‘diagnosis in the blink of an eye’

**DOI:** 10.1111/ene.16021

**Published:** 2023-08-13

**Authors:** Catarina Caldeiras, Edgar Seabra Sousa, Diogo Reis Carneiro, João Carvalho, Ricardo Rego, Ricardo Varela, Rui Araújo

**Affiliations:** ^1^ Neurology Department Centro Hospitalar Universitário São João Porto Portugal; ^2^ Department of Clinical Neurosciences and Mental Health, Faculty of Medicine University of Porto Porto Portugal; ^3^ Faculty of Medicine University of Porto Porto Portugal; ^4^ Neurology Department Centro Hospitalar Universitário de Coimbra Coimbra Portugal; ^5^ Faculty of Medicine University of Coimbra Coimbra Portugal; ^6^ Torrado da Silva's Centre for Child Development, Paediatrics Department, Hospital Garcia da Orta Almada Portugal; ^7^ Neurophysiology Unit Centro Hospitalar Universitário São João Porto Portugal; ^8^ Neurology Department Centro Hospitalar Universitário de Santo António Porto Portugal

**Keywords:** clinical neurology, clinical reasoning, diagnosis methods

## Abstract

**Background and purpose:**

The art of diagnosis in clinical neurology requires attentive listening and careful observation. In certain situations, a fragment of the history or a physical sign may be so distinctive that it allows clinicians to evoke a specific diagnosis. This quick mental process was previously referred to as ‘*Augenblickdiagnose*’ (‘diagnosis in the blink of an eye’) in a seminal paper by Dr. William Campbell in 1998. We aimed to revisit this concept by providing additional clinical vignettes.

**Methods:**

The authors wrote clinical vignettes using examples from their own clinical practice and performed a non‐systematic review of influential neurology textbooks using the words ‘pathognomonic’ and ‘highly suggestive’.

**Results:**

Twenty examples from various fields of neurology are presented in a table, stratified by major fields of neurology. A short educational reflection is provided for each diagnosis considered.

**Conclusion:**

‘*Augenblickdiagnose’* is an engaging teaching resource that also contributes to ‘neurophilia’, that is, a fascination for neurology, perhaps increasingly in today's modern neurology practice. However, multiple cognitive biases underlying mental shortcuts may lead to an incorrect diagnosis. It is important to stress that good clinical practice in neurology requires taking a thorough history and performing a careful neurological examination.

## INTRODUCTION

Neurology is a medical field where proficiency comes after several years of caring for persons with neurological disorders. Active and careful listening, strong observational skills, curiosity and intelligence are skills that serve the neurologist well. Parallel to this should be a profound knowledge of the structure and functioning of the nervous system and the clinical picture through which disorders manifest themselves [1, 2].

Making a diagnosis is most often not a linear process. In medicine, particularly in neurology, clinicians use data collected from the history, physical examination, and ancillary tests to generate working hypotheses. Then, the most plausible (or more alarming) diagnoses are considered first. In certain situations, a fragment of the clinical history or a physical sign may be so distinctive that it allows clinicians to evoke a specific diagnosis immediately. This quick mental process was previously referred to as ‘*Augenblickdiagnose*’ or ‘diagnosis in the blink of an eye’ in a seminal paper by Dr. William Campbell [3].

In this article, we revisit the concept of ‘*Augenblickdiagnose*’ by providing additional clinical vignettes, absent in the original paper. There are several reasons for doing this. First, the field of neurology has expanded significantly in the last 30 years, prompting new ‘classic’ presentations of relatively new disorders, such as anti‐N‐methyl‐D‐aspartate receptor (NMDAR) autoimmune encephalitis. Second, discussing a complex neurological condition or care plan, during ward rounds, demands scientific rigor, succinctness and clarity. Sometimes, critical decisions about the care plan are based on the clinician's summary (assuming the clinician has enough experience). This makes the recognition of major neurological syndromes and their narration as clinical vignettes a key component of teaching and learning in neurology. Finally, a large part of the appeal (or ‘*neurophilia*’) that draws students and junior doctors to neurology [4] may be attributed to the capacity to diagnose a ‘mysterious’ condition using one's observation (and listening) skills. ‘*Augenblickdiagnose*’ may contribute to that excitement.

## METHODS

The authors used their expertise, and vignettes were drawn based on people they have followed in their clinical practice. Additionally, they performed a non‐systematic review (using the ‘search’ command in the PDF reader) of the most recent editions of selected influential neurology textbooks, including *DeJong's The Neurological Examination* (8th edition) [5], *Bradley's Neurology in Clinical Practice* (8th edition) [6], *Adam and Victor's Principles of Neurology* (11th edition) [7] and *Neurology: a Queen Square Textbook* (2nd edition) [8] for the terms ‘pathognomonic’ or ‘highly suggestive’. The authors discussed the cases, and the most illustrative ones were included. The medical history and some personal characteristics have been altered to safeguard the privacy of the individuals described. Finally, the most important teaching points of the vignette are briefly discussed.

## RESULTS

We present 20 case vignettes grouped into a few subspecialties in neurology, namely, movement disorders, cognitive and neuropsychiatric disorders, neuromuscular disorders, epilepsy, headache and cerebrovascular disorders (Table 1).

**TABLE 1:** Case vignettes and diagnostic considerations.

Clinical vignette	Teaching points
Movement disorders	
A 41‐year‐old football referee complains of a ‘bad leg’. Several orthopaedic surgeons could not find anything wrong with him. He reported that he could run and walk backward much better than forward. His parents were first‐degree cousins. Examination revealed lower limb dystonia and asymmetrical parkinsonism	Running or walking backward better than forward should raise the possibility of lower‐limb dystonia. If parkinsonism is also present, notably if consanguinity is identified, consider genetic early‐onset Parkinson's disease (in particular autosomal recessive, e.g., PARK2 or PINK1). Also, repetitive dystonic episodes of the lower limbs occurring during physical exertion (in the absence of parkinsonism) should lead to the consideration of paroxysmal exercise‐induced dystonia
A 63‐year‐old man reports frequent falls. He had been diagnosed with depression recently, and his wife worries about his cognition. He is slow answering questions, has considerable rigidity in the neck, and has an apparent supranuclear vertical gaze palsy. He has parkinsonian features and low‐pitch dysarthria. Postural reflexes are markedly abnormal	The combination of early falls, axial rigidity, dysexecutive/frontal syndrome, dysarthria, and vertical gaze palsy in a patient with parkinsonism is highly suggestive of progressive supranuclear palsy. This is the classic presentation, known as Richardson syndrome. Parkinsonism is usually symmetrical, and tremor is not a prominent feature. Cervical dystonia with retrocollis is frequent. Patients show a distinctive surprised look, and the procerus sign is characteristic, i.e., forehead furrowing along with corrugator supercilii contraction. Apraxia of eyelid opening and blepharospasm might be present. Levodopa usually does not provide sustained clinical benefit
A 67‐year‐old woman complains of imbalance and a tremulous feeling in her legs, specifically when waiting in line in the supermarket and standing for long periods in church. Symptoms resolve once she resumes walking or sits down. She is otherwise well	A history of imbalance or tremor in the standing position that resolves when walking is highly suggestive of orthostatic tremor. Occasionally it is possible to hear the high‐frequency tremor using a stethoscope. Distinction from the much rarer orthostatic myoclonus requires electromyography
A 52‐year‐old man complains of gait impairment, cognitive dysfunction, and tremor. Previous medical history includes bilateral cataract surgery at age 40. Examination reveals cerebellar ataxia, postural tremor, and large masses in both ankles, which turned out to be xanthomas	The combination of cerebellar ataxia, cognitive impairment, tremor, and systemic features, i.e., cataract at a young age and tendon xanthomas is suggestive of cerebrotendinous xanthomathosis. This is an autosomal recessive leukodystrophy caused by pathogenic variants on the CYP27A1 gene, leading to accumulation of cholestanol and cholesterol. Hyperreflexia and seizures may also occur. Some additional clinical clues include neonatal jaundice, chronic diarrhoea, skeletal abnormalities, and premature atherosclerosis. Treatment with chenodeoxycholic acid may stabilize or even reverse some of the symptoms
Cognitive and neuropsychiatric disorders	
A 45‐year‐old man with a heavy history of alcohol abuse presents with a 1‐week history of confusion and imbalance. Examination reveals dysmnesia, ataxia and ophthalmoparesis with nystagmus	The combination of ataxia, ophthalmoparesis with nystagmus, and confusion or memory impairment in a patient with a history of significant alcohol abuse is suggestive of Wernicke's encephalopathy. It is caused by thiamine deficiency and may occur with poor intake or malabsorption. The complete neurological triad is not present in the majority of cases. Encephalopathy is the most frequent presenting symptom. Wernicke's encephalopathy is a medical emergency, and parenteral thiamine should be initiated as soon as possible
A 32‐year‐old woman with no prior psychiatric background presents with a 2‐week history of psychosis. Her family also reports two episodes of loss of consciousness. On examination, she is feverish, with poor attention, and displays irregular writhing movements of the upper limbs. In a few hours, she develops severe hypotension and respiratory failure, requiring intubation and admission to the intensive care unit	A psychiatric prodrome in a young woman followed by encephalopathy, a movement disorder, seizures and a mild fever, followed by severe autonomic failure is suggestive of an autoimmune disorder, specifically anti‐NMDAR encephalitis. An ovarian teratoma must be excluded in young women presenting with presumed autoimmune encephalitis. Prior herpes virus encephalitis is another well‐established trigger
A 68‐year‐old retired bank teller complains of progressive memory loss over the last 3 months. More recently he started having sudden, rhythmic jerks of the left face and arm	Rapidly progressive cognitive impairment associated with faciobrachial dystonic seizures is highly suggestive of autoimmune encephalitis mediated by antibodies against LGI1. Seizures are often refractory to antiseizure medication. Unexplained hyponatraemia and new‐onset REM behaviour disorder are additional clues
Neuromuscular disorders	
A 47‐year‐old man presents with a 4‐day history of blurry vision, dry mouth, muscular weakness and severe constipation. Examination revealed dilated non‐reactive pupils, ophthalmoparesis, facial palsy, flaccid tetraparesis and areflexia. Two additional family members are experiencing nausea and abdominal discomfort, after eating homemade smoked meats and sausages	Flaccid descending paralysis with cranial nerve impairment, associated with severe autonomic dysfunction following the ingestion of homemade sausages is suggestive of foodborne botulism. Wounds and intravenous drug use are another source of botulism
A 30‐year‐old woman is receiving treatment in the Haematology Department for leukaemia. Neurology is consulted because of new‐onset numbness in the chin area, confirmed by pinprick testing. Her neurological examination is otherwise normal	Sensory changes in the chin area, especially in the context of cancer, may be attributed to ‘numb‐chin syndrome’, a neoplastic infiltration of the inferior alveolar nerve or mental nerve. Although metastatic lesions in this region are rare, almost any type of tumour can cause numb‐chin syndrome. Even though it is classically associated with tumours, the most frequent causes are trauma and dental injuries
A 23‐year‐old man notices a dropped hand after waking up late on an uncomfortable couch on a Sunday. There is impaired sensation in the lateral dorsal region of the hand, and a hypoactive brachioradialis reflex. When he makes a fist, the hand drops even lower	A radial nerve neuropraxic injury (‘Saturday night palsy’) is one of the main causes of dropped hand, especially if history is positive for unergonomic positions and examination reveals sensory changes and abnormal reflexes. A cortical hand, i.e., a dropped hand in the setting of a cerebral lesion, specifically in the contralateral precentral gyrus, is an important differential diagnosis. In such cases, when making a fist, there may be synkinetic wrist extension instead of aggravated wrist drop, as illustrated here
Epilepsy	
A previously healthy 3‐year‐old boy started having staring spells lasting several seconds, especially early in the morning before breakfast. He had already been consulted by a paediatric neurologist because of a clumsy gait. Epilepsy was suspected and he started taking ethosuximide without clinical benefit. Of note, his mother had experienced bothersome ‘foot cramps’ after running since she was a teenager	Atypical absence seizures or atonic seizures starting very early in life can be due to a mutation in the SLC2A1 gene associated with GLUT1 deficiency syndrome. Atonic seizures, generalized tonic–clonic, seizures and hemiclonic seizures can also occur. It can be associated with microcephaly, developmental delay and ataxia. Epilepsy is usually refractory to antiseizure medications. Another possible presentation is paroxysmal exertion‐induced dyskinesia. Treatment with a ketogenic diet is indicated
A previously healthy 6‐year‐old boy woke up his parents in the middle of the night complaining of ‘feeling sick’. He started to vomit profusely. He was pale and his eyes were deviated to the right. He gradually became unresponsive and upon arrival at the Emergency Department, at least 15 min after symptom onset, he was still unresponsive and had persistent right conjugate eye deviation	Self‐limited epilepsy with autonomic seizures (Panayiotopoulos syndrome) should be suspected when a pre‐schooler presents with paroxysmal spells of dysautonomia, abdominal pain, nausea and vomiting, which arise from sleep. Vomiting is the most common and prominent feature, usually leading to misdiagnosis as a self‐limited gastrointestinal syndrome. The episodes are usually long, which correlates with non‐convulsive status epilepticus. Since the episodes are rare and the syndrome is self‐limited, antiseizure medication might not be required
An 87‐year‐old patient with type 2 diabetes mellitus is brought to the emergency department due to prolonged, involuntary, rhythmic jerks on one side of the body. He had stopped all medication, including insulin, because of financial constraints. Between jerks, he kept asking for water. Antiseizure medication administered in pre‐hospital emergency care failed to provide a significant effect	New‐onset epilepsia partialis continua in an elderly individual with diabetes should raise suspicion of an underlying hyperglycaemic hyperosmolar state, which may be a life‐threatening condition, demanding urgent metabolic correction and hydration. Blood glucose levels are usually extremely high and urinary ketones are absent. Seizure control can be difficult and requires correction of the underlying acute metabolic disorder
Headache disorders	
A 65‐year‐old woman presents in the Emergency Department for the fourth consecutive night due to a very intense headache occurring 2 h after sleep. Neurological examination is normal. She had given up her usual night‐time coffee prior to these episodes on the advice of a friend	After excluding more worrisome diagnoses such as a tumour, an aneurysm or vasculitis, a possible diagnosis for a headache that awakes a middle‐aged or elderly individual from sleep is hypnic headache, which may have been inadvertently treated by the usual night‐time coffee
A 44‐year‐old woman with a history of pituitary adenoma complains of a sudden and extremely violent headache associated with double vision. Her blood pressure is low and her pulse is fast. She is sleepy and presents a bitemporal visual field defect, impaired ocular movements and reduced sensation in the distribution of the trigeminal nerve on one side	An intense headache in a patient with pituitary adenoma (especially if loss of blood or hypotension occurs) with bitemporal hemianopsia and impaired oculomotor function (suggestive of chiasmatic and/or cavernous sinus involvement) should prompt the hypothesis of pituitary apoplexy. Acute hypopituitarism with adrenal insufficiency may cause life‐threatening hypotension
A 75‐year‐old man with hypermetropy experiences sudden intense ocular pain when the cinema screen goes black at the end of the movie. His vision is markedly reduced on the side of the pain, and the eyeball is engorged and tense	People with hypermetropy have a narrow ocular anterior chamber. When the pupil dilates (as occurs in the dark) this can precipitate closed‐angle glaucoma causing a sharp pain in the eye, which can become red and tense. This may be mistaken for trigeminal autonomic cephalalgia
A young woman taking a combined oral contraceptive pill reports an intensely diffuse headache in the last 3 days. The headache worsens when lying down and coughing. She also has pulsatile tinnitus, nausea, and transient double vision when looking sideways	A young woman taking a contraceptive pill that presents a new‐onset severe headache with features of intracranial hypertension should lead to the consideration of cerebral venous thrombosis. Seizures and neurological deficits can also occur. Headache is the most common feature. There may be no signs of intracranial hypertension on examination, e.g., papilloedema, therefore, a CT or MRI venogram should be performed in all suspected cases
Stroke and vascular disorders	
A 65‐year‐old overweight man with a history of cigarette smoking complains of recurrent episodes of vertigo associated with pain and numbing of his left arm. These episodes are triggered by repetitive movements of his arm. His left radial pulse is weak with blood pressure of 120/50 mmHg on the left arm, while it is 190/90 mmHg on the right. He has small ulcers on his fingertips	The presence of transitory dysfunction of the brainstem or the cerebellum triggered by brachial exercise in a patient with signs of peripheral artery disease is suggestive of subclavian steal syndrome. Atheroembolism may lead to livedo reticularis, digital ischaemia, small distal ulcers, and splinter haemorrhages under the nail bed. A significant difference in upper extremity blood pressure is an important clinical feature
A 69‐year‐old taxi driver with degenerative cervical spine disease complains of several episodes of vertigo and double vision when looking behind his shoulder while parking the car	‘Bow‐hunter's syndrome’ is a rare cause of stroke or, more frequently, transient ischaemic attack of the vertebrobasilar circulation due to compression of the vertebral artery during rotation of the neck, usually because of osteophytes or bone spurs
A 70‐year‐old woman with a history of hypertension suddenly develops erratic dancing movements on one side of her body, compatible with hemichorea.	In the absence of hyperglycaemia, a stroke is probably the cause of newly‐onset sudden hemichorea in the elderly. Hemiballismus may also be present. In the past, it was considered pathognomonic of subthalamic nucleus infarction, but it can also occur with infarction of the caudate nucleus, globus pallidus and the surrounding white matter

Abbreviations: CT, computed tomography; GLUT1, glucose transporter 1; LGI1, leucin‐rich glioma inactivated 1; MRI, magnetic resonance imaging; NMDAR, N‐methyl‐D‐aspartate receptor; REM, rapid eye movement.

## DISCUSSION

Diagnosis in clinical neurology is intellectually stimulating detective work [9]. The examples provided represent clinical scenarios where an aspect of the history or examination is so distinctive that it should trigger a diagnostic rationale immediately, even without additional information. It is important to stress that the table presented is not definitive nor all‐inclusive. The authors mainly focused on their areas of expertise and welcome further input from colleagues working in other fields.

Moreover, we do not intend to suggest that neurological diagnoses should be made carelessly with haste. A precise diagnosis in neurology requires a complete and thorough history and physical examination, as well as a careful weighing of pros and cons regarding each working hypothesis. In an era where ‘Googling’ medical symptoms is common practice, an analogy can be made with ‘*Augenblickdiagnose*’: if only a small piece of information is entered into the search engine by someone who does not have the skills to properly weigh the results, an endless list of seemingly equally valid diagnoses will emerge. On the other hand, if the technology is employed by a seasoned clinician with years of experience, it is likely that the information will be adequately filtered and applied correctly.

Skilled physicians use distinct types of clinical reasoning to achieve a medical diagnosis [10]. In situations where the pattern is immediately identifiable as highly suggestive of a certain condition, they will quickly capture the global impression, that is, the ‘*gestalt*’ of the case [11], and use fast and intuitive logic, also known as type 1 processing. [12] This approach, of which ‘*Augenblickdiagnose*’ may be the most extreme example, is less time‐consuming and is effective in most straightforward cases. However, it is important to stress that mental shortcuts such as ‘*Augenblickdiagnose’* may sometimes lead to misdiagnosis. This may happen, for example, when physicians ‘zoom in’ in one small aspect, while disregarding the whole clinical picture. [13] This phenomenon was dubbed ‘tunnel vision’, which occurs when skilled neurologists consider diagnoses primarily within their field of expertise [14]. Conversely, a slower, more time‐consuming and systematic reasoning is employed when a pattern is not identified immediately, also known as an analytical approach or type 2 processing [12]. In their clinical practice, physicians often switch between different approaches [12].

In the ‘eyes’ of skilled physicians, the usefulness of ‘*Augenblickdiagnose*’ should not be disregarded. However, as explained above, it is critical to be aware of the cognitive biases that may flaw this reasoning, and which situation requires the use of different diagnostic strategies. Importantly, a functional magnetic resonance imaging study of experienced clinical neurologists managing straightforward and more ambiguous cases showed differences in brain connectivity between the two types of situation. The authors propose a dynamic model of medical reasoning, where the ‘schematic anticipation’ of the consequences of reaching a correct diagnosis is the starting point, and the readiness to attain a result depends on the gap between the anticipated and the final solution and the information needed to fulfil that gap [15].

Independent of the limitations of each mental process, the recognition of precious clinical clues cannot be missed, regardless of how trivial they may appear at first glance (‘my work is based on the observation of trifles’, fictional detective Sherlock Holmes famously said). [16] Furthermore, learning by clinical vignettes is important to junior doctors and medical students as it enhances clinical acumen and narration skills. ‘*Augenblickdiagnose’* appeals to an intangible trait of neurology, in which light is shed on an obscure problem by asking the right question or by performing the most suitable diagnostic gesture. We hope this paper contributes to that fascination.

## AUTHOR CONTRIBUTIONS

Catarina Caldeiras, Diogo Carneiro, João Carvalho, Ricardo Rego, Ricardo Varela and Rui Araújo all provided cases inspired by patients they have encountered in their clinical practice. Catarina Caldeiras and Edgar Seabra Sousa contributed significantly to early versions of the manuscript. Diogo Carneiro, Ricardo Rego and Ricardo Varela provided significant input to later versions of the manuscript. Rui Araújo provided the original concept and significantly contributed to the manuscript's early and later versions.

## FUNDING INFORMATION

The authors confirm that this study received no funding.

## CONFLICT OF INTEREST STATEMENT

None of the authors has competing interests to declare.

## Data Availability

The data that support the findings of this study are available from the corresponding author upon reasonable request.
